# Temporal genomics reveal rapid adaptation to pesticide exposure in Eastern honeybees

**DOI:** 10.1093/nsr/nwaf438

**Published:** 2025-10-15

**Authors:** Shanlin Liu, Lifei Qiu, Danlei Liang, Agnièle Touret Alby, Mikkel-Holger S Sinding, Min Tang, Mohd Zulkifli Mustafa, Faezah Msalleh, Chunsheng Hou, M Thomas P Gilbert, Xin Zhou

**Affiliations:** State Key Laboratory of Animal Biodiversity Conservation and Integrated Pest Management, Institute of Zoology, Chinese Academy of Sciences, China; State Key Laboratory of Agricultural and Forestry Biosecurity, MOA Key Lab of Pest Monitoring and Green Management, College of Plant Protection, China Agricultural University, China; Cardiff University – Institute of Zoology Joint Laboratory for Biocomplexity Research, Chinese Academy of Sciences, China; State Key Laboratory of Animal Biodiversity Conservation and Integrated Pest Management, Institute of Zoology, Chinese Academy of Sciences, China; State Key Laboratory of Agricultural and Forestry Biosecurity, MOA Key Lab of Pest Monitoring and Green Management, College of Plant Protection, China Agricultural University, China; Muséum National d’Histoire Naturelle (MNHN), France; Center for Evolutionary Hologenomics, The GLOBE Institute, University of Copenhagen, Denmark; Department of Biosciences and Bioinformatics, School of Science, Xi’an Jiaotong-Liverpool University, China; Department of Neuroscience, School of Medical Sciences, Universiti Sains Malaysia, Kubang Kerian, Malaysia; Department of Biosciences, Faculty of Science, University Teknologi Malaysia (UTM), Malaysia; Institute of Bast Fiber Crops, Chinese Academy of Agricultural Sciences, China; Center for Evolutionary Hologenomics, The GLOBE Institute, University of Copenhagen, Denmark; University Museum, Norwegian University of Science and Technology (NTNU), Norway; State Key Laboratory of Agricultural and Forestry Biosecurity, MOA Key Lab of Pest Monitoring and Green Management, College of Plant Protection, China Agricultural University, China

The Eastern honeybee (*Apis cerana*) is a major insect pollinator of both agricultural and forestry ecosystems in Asia, providing pollination services that benefit half of the world’s population [1]. Facing numerous threats, the habitat of *A. cerana* has experienced significant reduction, with most of their populations restricted to rural regions [2]. To what extent these range changes have altered honeybee genetic traits remains elusive. Inspired by this, we generated temporally and geographically distributed genome data from 46 historical samples of Eastern honeybees dated to ca. 120 years ago (Fig. 1A, Table S1), to provide direct estimates of population genetic responses to anthropogenic changes during the last century. A total of 362 modern *A. cerana* samples were collected from across temperate and tropical Asia. The sampling was strategically designed to cover major distinct genetic units, representing nine geographic populations (Fig. S1, Table S2), as revealed by our previous work [3,4]. Genome alignment showed that most of the museum samples yielded high levels of endogenous DNA (>50%), although in a fragmented state, with a median read length of ∼40 bp (Fig. S2). Based on population structures inferred from the identity-by-state (IBS) distance matrix, the introduction of historical individuals into the modern dataset did not reveal any new genetically distinct clusters (Fig. S3). These results showed that no historical lineage has been lost over the past 100 years and most historical individuals clustered together with the central population, aligning well with their geographic origin.

**Figure 1.**
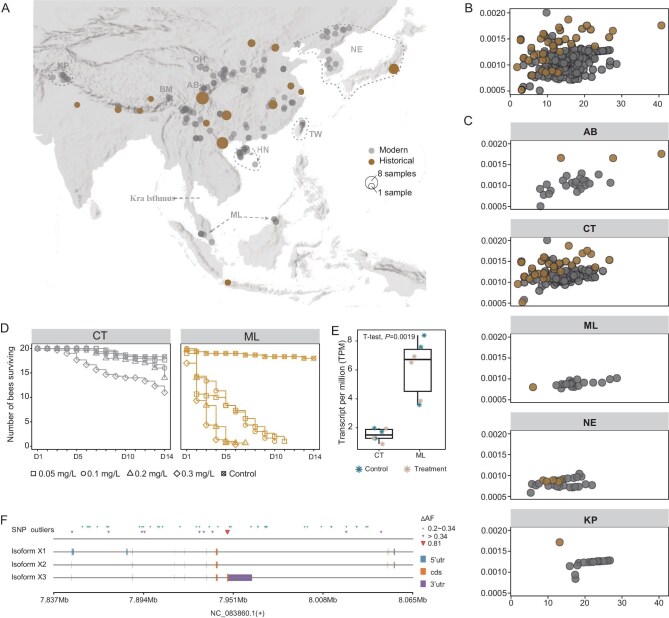
Genomic changes in honeybees revealed by museomics. (A) The geographical distribution of modern and historical honeybee samples. The locations of the historical samples are indicated by orange dots, scaled to indicate sample numbers. Gray dots represent modern samples. As latitude and longitude details were unavailable, the locations of the historical samples on the map are represented by the capital city or township based on collection information provided on the voucher specimens. Populations are labelled as follows: Aba, AB; Bome, BM; Central, CT; Hainan, HN; Malaysia, ML; Northeast, NE; Kashmir & Pakistan, KP; Qinghai, QH; Taiwan, TW. (B and C) Genetic diversity (genome heterozygosity, *y*-axis) and depth of genome coverage (*x*-axis) of the honeybees. (B) includes all samples, while (C) categorizes them into different genetic populations. Historical and modern samples are color-coded as indicated in the figure legend. Note that the heterozygosity estimates are based solely on transversions to avoid artificial inflation derived from post-mortem deamination of cytosines in historical samples. The current plot includes only the populations of AB, NE, KP, CT and ML, as historical samples from TW, HN, BM and QH were unavailable. (D) The survival of honeybee workers after being exposed to imidacloprid. The *y*-axis represents the average number of surviving individuals across three biological replicates. Results of each biological replicate are detailed in Fig. S8. The survival rate in the imidacloprid exposure test between the central China and Malaysia populations differs significantly (*P* value < 2 × 10^−^^16^, log rank test) in all but the control group (*P* value = 0.7). (E) Gene expression profile of *nAChR* α1 isoform X3. Individuals from the control group (feed without imidacloprid) and the treatment group (feed with 0.05 mg/L imidacloprid) can be distinguished by their corresponding colors. (F) Genetic variants on *nAChR* α1. SNPs are represented by triangles to indicate their corresponding locations and AF differences across time (by size). Review drawing number: GS 京(2025)2413号.

We estimated genome-wide heterozygosity (*Ho*) for each individual following the method of Liu *et al.* [5]. Given that the historic data derive from pinned museum specimens collected ∼120 years ago, their DNA contains cytosine deamination damage signatures (Fig. S2), which could lead to an artificially elevated transition signal [5]. Thus *Ho* was estimated using transversions only, and revealed that genomic heterozygosity was, overall, relatively higher in the historical samples than in the modern samples (Fig. 1B). However, subdivision of the modern samples into their corresponding populations revealed that not all populations have experienced a reduction in genetic diversity. Specifically, while genetic diversity appears to have been lost in the Aba, Central and Kashmir & Pakistan populations (*P* value < 0.01 for Aba and Central; Table S3), this is not the case in the Malaysia and Northeast populations (Fig. 1C). We do caution, however, that the small sample sizes of the historical Malaysia, and Kashmir & Pakistan populations may need further investigation, to confirm if this is the case (only one historical individual was included in the dataset for each). Additional sampling of museum samples would help clarify

the cause of the clear difference observed between the historical and modern individuals from the Kashmir & Pakistan population.

To identify the genomic changes that have accompanied the genetic reduction revealed in our analysis, we applied both cross-population composite likelihood ratio (XP-CLR) and cross-population extended haplotype homozygosity (XP-EHH) approaches to detect selective sweeps by comparing the central populations from two different timepoints (detailed in the Supplementary Data). This analysis yielded 69 common genes, with 54 of them being protein-coding genes (Table S4). Functional enrichment analysis of these genes showed that Gene Ontology (GO) terms related to the nervous system were significantly enriched, including: cellular components of the synaptic membrane—ion channel complex; biological processes of response to chemicals—neuron projection guidance; and molecular functions of nicotinergic acetylcholine receptor activity (*nAChR*) and signaling receptor activity (Fig. S4A, Table S5). Ion channels within the insect nervous system are the primary targets of most commercial insecticides; we therefore speculated that pesticides could have acted as a major evolutionary force that shaped the genomic landscape changes of Eastern honeybees over the last century.

We further extracted the single nucleotide polymorphism (SNP) outliers whose allelic frequencies (AF) have shifted dramatically during the last century (top 1% and a corresponding ΔAF ≥ 0.34), resulting in a set of 3958 SNPs. Intriguingly, for those SNP outliers, we noticed that the modern Malaysia population, in contrast to the other peripheral populations, exhibited a significantly lower AF variance when compared to the historical central population (detailed in Fig. S5). This suggests there is a greater similarity between the historical central population and the modern Malaysia population in the gene regions where those SNP outliers are located. This heightened genetic affinity was further supported by outgroup *F3* statistics (Fig. S6), a population genetic method quantifying shared drift [6]. Given that demographic processes typically generate genome-wide signatures, the anomalous AF variance patterns in Malaysia were restricted to outlier SNPs, suggesting that the selective force acting on these sites in Malaysia was weaker or less pervasive. Furthermore, despite the relatively small sample size, the overall genetic diversity is well captured by the Malaysia genomic data (Fig. S7), thus precluding sampling bias as a major concern. Based on the findings that the functional enrichment of genes associated with the outlier SNPs demonstrated patterns consistent with those detected in the previous selection analysis (Fig. S4B), we therefore interpret this as a potential indication that honeybees from contemporary Malaysia may have experienced lower selective pressure from toxic pesticides compared to those from central China.

To test our hypothesis, we explored whether there is a difference between modern honeybees from Malaysia and central China, in terms of resistance to a neonicotinoid insecticide (imidacloprid). Our results showed that the survival rate of the modern central China population was significantly higher than that of the modern Malaysia population (*P* value < 2 × 10^−^^16^) after being exposed to imidacloprid for 14 days (Fig. 1D). We also observed that survival rates in central Chinese bees were not significantly affected by imidacloprid exposure until exposed to a concentration of >0.1 mg/L, after which detrimental effects began to occur after ca. 5 days’ exposure. In contrast, the viability of bees from Malaysia was immediately negatively influenced after being exposed to imidacloprid, even when exposed to the lowest concentration tested (0.05 mL/L) (Fig. 1D, Table S6). These results match the fact that, while neonicotinoids have been used in both Malaysia and China over the past 100 years, much higher doses and application frequencies have been applied in the latter, as documented by surveys of neonicotinoids in honey, water and sediments [7,8].

We further inspected the expression profiles in the two honeybee populations to understand the evolutionary mechanisms underpinning the variation of pesticide resistance observed between the two populations. Among these rapidly selected genes, we observed significantly lower expression of *nAChR α1* isoform X3 in the China central population, in comparison with the Malaysia population (Fig. 1E). The *nAChR α1* gene, encoding a key component of the nAChR complex, was consistently detected to have undergone adaptive evolution in both XP-CLR and XP-EHH analyses. This gene was also enriched with SNP outliers, including 10 SNP outliers (ΔAF ≥ 0.34) and dozens of SNPs with a rapid change in AF (ΔAF > 2 SD.). The majority of these were located in introns, while three were specifically found in untranslated regions (UTRs)—one on the 5′-UTR of isoform X1 and two on the 3′-UTR of isoform X3 (Fig. 1F). The 3′-UTRs contain a particularly important class of non-coding variants, which can impact post-transcriptional and translational processes [9], and a low expression level of *nAChR* subunits is associated with neonicotinoid resistance in insects [10]. Therefore we suggest that genetic variants on *nAChR α1*, especially those located near the 3′-UTR of isoform X3, could have played an important role in determining the varied resistance to imidacloprid observed between the central China and Malaysia populations.

In summary, based on the genetic diversity observed in historical samples, we found that, even over the relatively short time span of one century, the genetic composition of several evolutionary lineages of the Eastern honeybee has changed significantly. For example, the central population has experienced a notable reduction in genetic diversity, despite remaining widely distributed. This trend sounds a serious alarm for the fate of the species, as *A. cerana* has followed a centrifugal diversification model in its recent evolutionary history, where the central population has served as a genetic source, from which descendent lineages derive and eventually become adapted to novel ecological niches [3,4]. Even if population numbers are restored, the lost genetic variation may be difficult to recover, or worse, genetic diversity may continue to erode due to genetic drift and the lingering effects of past population bottlenecks. It suggests that effective conservation strategies are desperately needed, as the decline of genetic diversity within its core range jeopardizes the evolutionary potential and adaptive capacity of *A. cerana* in the face of future environmental challenges. To prevent further genetic erosion, it is essential to uphold integrated pest management principles and minimize bee exposure to pesticides. Additionally, effective conservation of insect pollinators requires accurate identification of the causes of their decline, followed by appropriate mitigation efforts. Other pollinators, including bumble bees and solitary bees, face similar selection pressures and require further investigation to determine whether they have undergone rapid evolution or are being pushed toward extinction.

## Supplementary Material

nwaf438_Supplemental_Files

## Data Availability

The newly sequenced data have been archived at the NCBI SRA under Accession No. PRJNA1169767.
