# Supplementary material for: Temporal genomics reveal rapid adaptation to pesticide exposure in Eastern honeybees
Source: Natl Sci Rev. 2025 Oct 15;13(12):nwaf438. doi: 10.1093/nsr/nwaf438 (PMC13317446; doi:10.1093/nsr/nwaf438)
Supplement: nwaf438_Supplemental_Files [file nwaf438_supplemental_files.zip › NSR supplementary data_1012.docx]

**Supplementary Data**

Temporal genomics reveal rapid adaptation to pesticide exposure in Eastern honeybees

Shanlin Liu^1,2,3,^*, Lifei Qiu^1^, Danlei Liang^2^, Agnièle Touret Alby^4^, Mikkel-Holger S. Sinding^5^, Min Tang^6^, Mohd Zulkifli Mustafa^7^, Faezah Msalleh^8^, Chunsheng Hou^9^, M. Thomas P. Gilbert^5,10,^*, Xin Zhou^2,^*

***Corresponding authors (Emails):**

[shanlin.liu@ioz.ac.cn](mailto:shanlin.liu@ioz.ac.cn)

[xinzhou@cau.edu.cn](mailto:xinzhou@cau.edu.cn)

[tgilbert@sund.ku.dk](mailto:tgilbert@sund.ku.dk)

**This document contains:**

Supplementary Materials and Methods

Supplementary Figures S1-S10

Supplementary References 1-30

**Supplementary Materials and Methods**

**Sample preparation and sequencing**

For the museum samples, we removed a single hind leg from each specimen using sterile plastic forceps. To protect the external structure of the removed leg, DNA was extracted using a “minimally destructive” method [1, 2]. Both the DNA extraction and subsequent library preparation were carried out in a dedicated PCR-free clean laboratory designed for aDNA work. First, we removed external contaminants from each sample using a 5% bleach wash, followed by a wash with 100% ethanol and subsequent two washes using molecular-grade water. We then added 300 μl digestion buffer to each sample and incubated them at 56°C and 350 RPM overnight (> 20 hours). The samples were then purified at a 1:10 ratio with a modified binding buffer using 5 µg Monarch DNA clean-up columns (New England Biolabs). After that, each extract was eluted in 42 μl of Elution Buffer (Qiagen) containing 0.05% TWEEN20 (Sigma-Aldrich) and 21 μl EBT. The elution step was repeated for each sample to maximize DNA yield. We built sequencing libraries for DNA samples following the SCR protocol [3] in which the recommended amounts of adapters and single-stranded binding proteins (SSB) were prepared for each DNA sample according to a DNA input tier system. Libraries were purified using the MinElute reaction clean-up kit (Qiagen) and eluted in 30 μl EBT. Five μl of each DNA library template was used for indexing PCR amplification following a PFU Turbo CX protocol at 14 cycles (see Table S7 for reagent and thermal cycling details). Amplified libraries were purified with HiPrep MagBio beads (Magbio Genomics) at a 1.2x ratio. The DNA libraries were sent for genome sequencing using an Illumina Nova-Seq platform with a strategy of paired end 150 bp. For the modern samples, genome sequences were obtained from our previous study (Ji et al. [4] and Qiu et al. [5], detailed in Table S2). Briefly, genomic DNA was extracted from the head and thorax of each worker bee using the DNeasy Blood and Tissue Kit (Qiagen, Germany) following the manufacturer’s instructions or using the phenol-chloroform extraction method. The DNA extracts were then sequenced on a BGISEQ-500 platform with a sequencing strategy of 200 to 400 bp insert libraries, 100-bp paired-end sequencing, and 3.5 to 5 Gb raw data per sample (>10X coverage).

**Genome mapping and genotyping**

We merged the paired-end reads using a modified version of AdapterRemoval v2.3.1 [6], which masked the conflict bases that have identical sequencing qualities as Ns and removed collapsed reads < 30 bp. The collapsed reads were mapped onto a genome assembly of *A. cerana* (NCBI accession number: PRJNA1169767, Table S8) using the BWA-ALN algorithm v0.7.17 [7] with disabled read seeding (-l 1024) to enhance error tolerance. For the modern samples, paired-end reads were quality-controlled using Fastp 0.13.1 [8] with parameters “-q 10 -n 10 -u 40” and mapped onto the same genome assembly using BWA ALN v0.7.17 [7] with PCR duplications masked using SamBamba [9].

After genotyping using the GATK v4.1.4.0 pipeline [10], we retained only biallelic sites for which more than 70% samples can be successfully genotyped and reach a genotype quality (GQ) ≥ 30. Furthermore, to mitigate the influence from DNA damage, we trimmed 5 bp from the 5' end of each read, and further masked the genotypes as missing for SNPs exhibiting transitions(C->T, G->A) which have a read number support < 2, or < 1/5 of the total depth.

**Genome reference**

We generated the reference genome using a dataset comprising three types of reads: Nanopore ultralong reads, Pacbio HiFi long reads and MGISEQ shotgun reads (Table S8). First, we assembled the Nanopore long reads into a draft genome using NextDenovo v2.1-beta.0 [11] with the following parameter setting: read_cutoff = 1k, seed_cutoff = 30k, and blocksize = 1g. To correct potential errors derived from the Nanopore long reads, we polished the assembled contigs using the HiFi long reads with Racon v1.4.13 (https://github.com/isovic/racon). Further refinement was achieved through two rounds of genome polishing with the shotgun reads using NextPolish v1.3.0 [12]. Finally, we evaluated the completeness of the genome using BUSCO v4.1.4 [13] with the hymenoptera_odb10 dataset.

**Data processing and population genetics**

We first removed SNP sites in strong linkage disequilibrium using PLINK (v1.9) [14] with a window size of 50 kb, a step size of every 5 markers and an r^2^ threshold of 0.5 (--indep-pairwise 50 5 0.5). Next, kinship coefficients were estimated separately for the modern and museum samples to identify family relationships using KING (v2.1.6) [15]. Only unrelated individuals (kinship > 2nd-degree) were kept for subsequent analyses. We also removed variant sites that exhibited excess heterozygosity from further analyses. Those sites likely resulted from incorrect mapping of reads from homologous genome regions and consistently showed abnormal depth of coverage (Fig. S9).

We calculated the identity-by-state (IBS) distance matrix and allele frequencies for each population based on whole-genome SNPs using PLINK (v1.9) [14] The IBS matrix was used for genetic structure estimation with a mutual k-nearest neighbor graph method as implemented in NetView [16], and the Nonmetric Multidimensional Scaling (NMDS) configuration function of *metaMDS* - an R package implemented in *vegan* [17].

The genome coverage varies across sampled populations, with the historical samples being consistently lower. Insufficient genome sequencing may cause errors in base-pair calling (hard genotype calling). To avoid this, we estimated genome-wide heterozygosity for all honey bee individuals following Liu et al. [18]. Briefly, this method is based on the Site Frequency Spectrum, where one diploid individual will generate three allelic states: homozygous ancestral allele state (AA), heterozygous state (AB), and homozygous derived allele state (BB). As such, the whole genome level heterozygosity rate can be calculated as AB/(AA+AB+BB). We applied the doSaf function (-doSaf 1) module in ANGSD v0.924 [19] to calculate genome-wide heterozygosity for each individual with the following parameters: unique mapping, a minimum mapping quality of 20, a minimum base quality of 20, a minimum and maximum depth values of 1/3 and 2 times of the average genome depth, respectively. By avoiding hard genotype calls, ANGSD works with genotype likelihoods (GLs), reducing biases that can arise from miscalling heterozygous sites as homozygous and has thus been widely adopted in genomic analyses of low-coverage data [19]. In addition, transition sites were removed (-noTrans 1) to alleviate the potential influences of DNA damage caused by cytosine deamination. This particular type of post-mortem DNA damage, frequently observed in historical samples, can artificially inflate genetic diversity estimates [18].

**Adaptive selection**

We applied XP-CLR (Cross-Population Composite Likelihood Ratio) [20] and XP-EHH (Cross-Population Extended Haplotype Homozygosity) [21] to identify gene candidates that have recently undergone selective adaptation. The two methods are robust and frequently employed statistical approaches for identifying selective sweeps, especially when comparing two populations. The two methods leverage distinct aspects of genetic variation: XP-CLR evaluates extreme and rapid allele frequency changes that are unlikely due to random drift, by considering multi-locus genotype frequencies across a window, while XP-EHH measures the age of alleles based on the extent of Linkage Disequilibrium (LD) around it.

**Gene ontology (GO) enrichment**

We applied Bedtools [22] to obtain the protein-coding genes overlapping the SNP outliers. These genes were subsequently projected to their *Drosophila melanogaster* orthologs. Gene ontology (GO) enrichment analysis was performed on these orthologs using ShinyGO v0.741 [23].

**Assessment of population structure based on SNPs that shift rapidly across time**

We calculated SNP allelic frequencies for each population using PLINK v1.9 [14] and summarized to capture their distribution features, and to identify outliers exhibiting pronounced temporal shifts in allele frequency in the mainland China central population, given that only the central population contained sufficient historical samples to robustly analyze allele frequency changes over time. In general, allelic frequency across time has not changed strongly for most of the SNPs, following a normal distribution with a mean of zero (Fig. S10). We applied a proportion threshold of 1% (about three standard deviations above the mean value and a corresponding allelic frequency change of 0.34) to determine SNPs whose allelic frequencies have changed significantly across the two time points. As the SNP outliers detected in this population reflect temporal changes specific to the central population itself, we calculated allele frequency variance (AFV) for each SNP outlier between the eight peripheral populations and the historical/modern central population using a formula: AFV = $\left| {AF}_{X}-{AF}_{Y} \right|$, where AF denotes allele frequency, and _X_ represents the modern or the historical central population, while _Y_ represents each of the peripheral populations. Thus, for each peripheral population we derived two AFV metrics: AFV _Modern_ = $\left| {AF}_{modern}-{AF}_{Y} \right|$ and AFV _History_ = $\left| {AF}_{history}-{AF}_{Y} \right|$. In this context, a smaller AFV indicates higher similarity in allelic frequency. These SNPs may have also undergone rapid temporal changes in each peripheral population and thus exhibit patterns where each population is more closely related to the modern central population than to the historical central population (AFV _Modern_ > AFV _History_), or conversely, show AFV _History_ > AFV _Modern_ and thus being in the historical state. In addition, we also conducted the outgroup *F3* statistics for the outlier SNPs using POPSTATS, a python program for computing f-statistics [24].

**Toxicology experiments for different Eastern honey bee populations**

We performed toxicology experiments for both the central population and the Malaysian population, to validate the potential rapid adaptation of the central population in terms of resistance to insecticide. We used imidacloprid (CAS: 138261-41-3, Shanghai Aladdin Biochemistry Technology Co.), a typical neonicotinoid that has been widely used in mainland China since the 1990s, to feed honey bee individuals and examine the mortality differences between the two populations.

We collected around 240 honeybee individuals in Yunnan China and Malaysia, respectively, to represent the CT and ML population. To ensure optimal health status of the bees used for the experiment, colonies were selected based on the following criteria: standardized colony size (assessed by nest and comb counts), randomized hive sampling, experimental timing restricted to typical active seasons (avoiding overwintering or peak summer phases), and the absence of observable disease indicators (e.g., parasites, pathogens, or microbial infections). Both populations were subjected to the same experiment design as follows: workers were fed with a 50% sucrose solution for 7 days after their emergence, followed by either the same sucrose solution (control group) or an imidacloprid-treated sucrose solution (exposure groups), for the subsequent 14 days. imidacloprid was dissolved in 50% sucrose solutions to prepare treatment solutions at gradient concentrations of 0.05, 0.1, 0.2 and 0.3mg/L. The sucrose solutions used for the experiment were freshly prepared and renewed daily. Each treatment includes 60 individuals that have been further divided into three independent biological replicates (20 individuals per replicate).

All the experiments were conducted in incubators with a constant temperature of 34.5±0.5℃, and apparatus used in the experiments, like cages, tweezers, etc., were sterilized by autoclaving prior to use.

We performed the Log-rank test to determine the survival rate differences between the honeybees from central China and Malaysia using the R package ‘survival’ [25].

For each group, we included 10 extra honeybee individuals, of which three bee individuals were randomly selected in the middle of the experiment (the 7th day) and were instantly put in liquid nitrogen for preservation. For each sample, the entire abdomen, including both fat body and the gut tissue, was used for RNA sequencing to reveal the overall insecticide responses of the honeybees. As the survival rate of the Malaysia bee individuals fed with imidacloprid was too low to sustain the population to the 7th day, only the control group and the treatment with lowest concentration of imidacloprid (0.05 mg/L) were sampled for RNA sequencing. The samples were sequenced using local sequencing providers - Novagene China and NextGene Malaysia, respectively. We mapped the sequencing reads of mRNA onto the recently updated genome (Acer_k1.0, GCF_029169275.1) using STAR (v2.7.0e) [26] and applied StringTie v2.2.3 [27] for gene and transcript quantification. Differential Expressed Genes (DEGs) were identified between groups using DEseq2 (v4.2.1) [28]. Genome annotations and genetic variation coordinates were converted between different genome assemblies using CrossMap v0.6.5 [29].

**Supplementary figures**


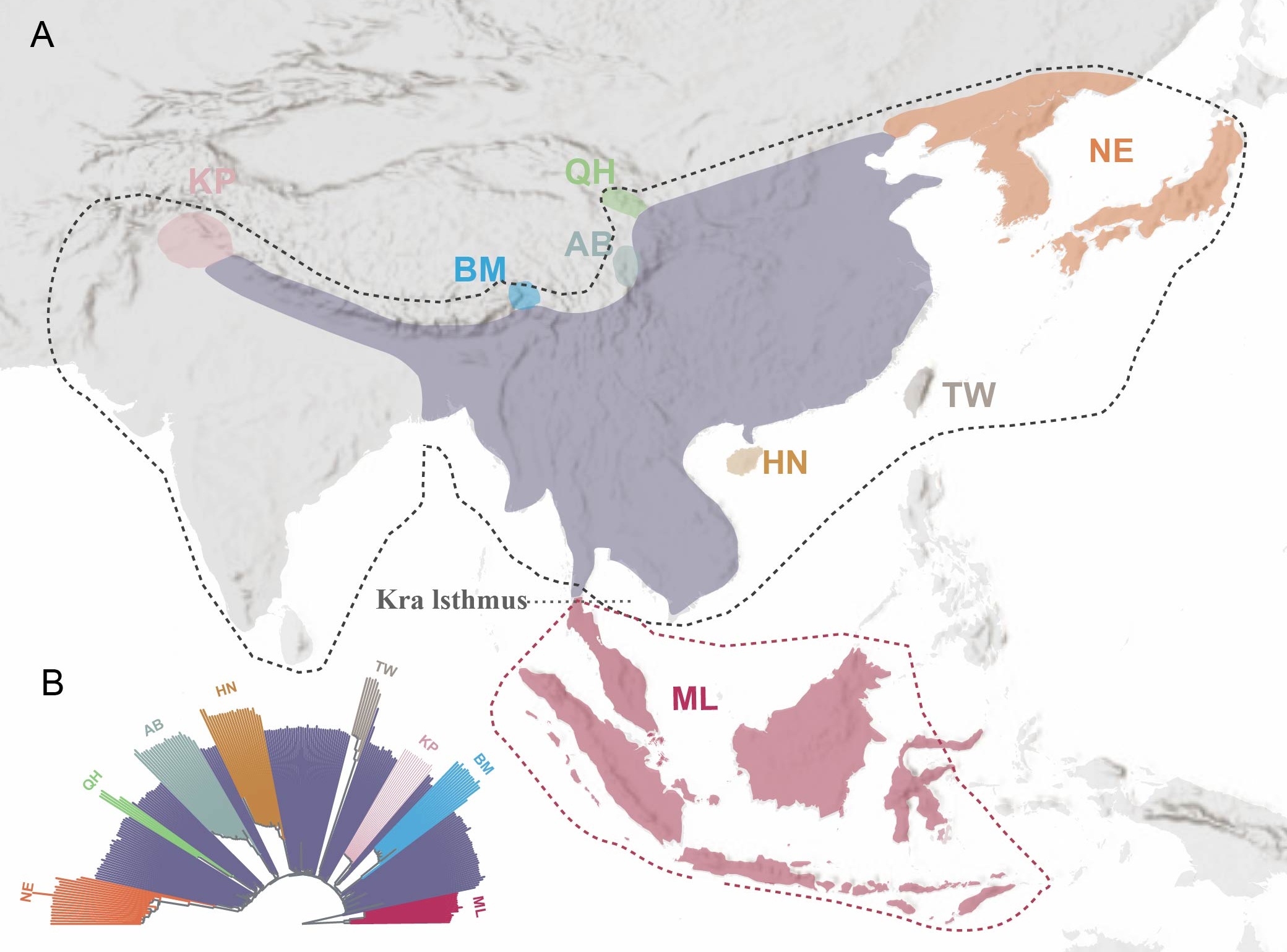


**Figure S1. Population structure and phylogenetic relationships of the eastern honey bee, *A. cerana.*** (A) native ranges of eight populations constituting the mainland lineage of *A. cerana*, and the Sundaland lineage. (B) phylogenetic tree constructed from nuclear SNPs, illustrating the monophyly of seven peripheral descendant populations and the Malaysian outgroup. Populations are labelled as follows: AB (Aba), BM (Bome), CT (Central), HN (Hainan), ML (Malaysia), NE (Northeast), KP (Kashmir & Pakistan), QH (Qinghai), and TW (Taiwan). The figure was modified from Qiu et al. [5].


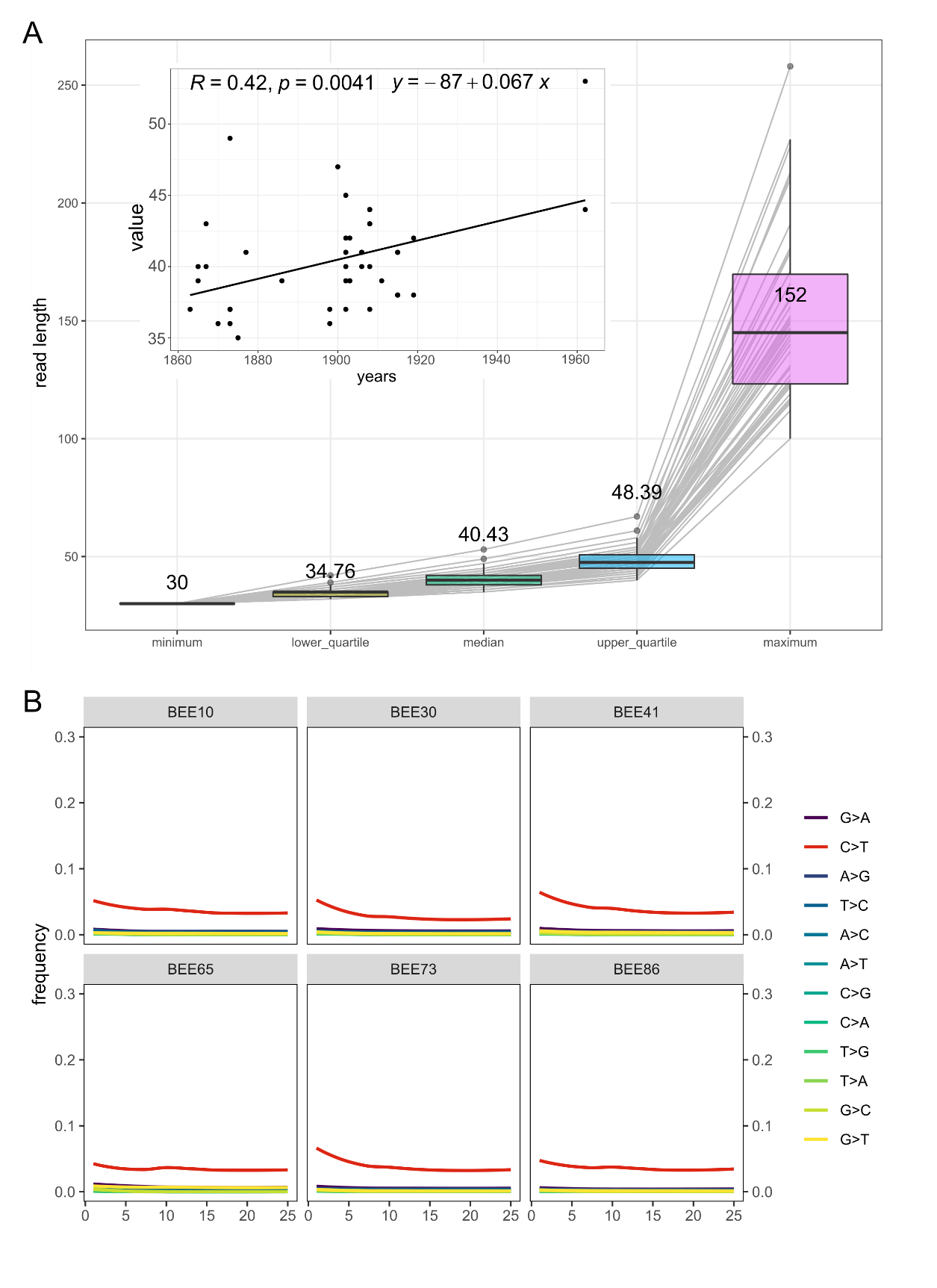


**Figure S2. DNA fragment lengths for the historical honey bee samples (A) and map damage plot for six randomly selected samples (B).** (A) the minimum, lower quartile, median, upper quartile, and maximum read length (X-axis) were calculated for each sample and summarized in a box plot. The inset in the top left corner illustrates a positive correlation between read length and collection date. (B) the x-axis denotes positions on reads and the y-axis denotes frequencies for all kinds of mutation types.


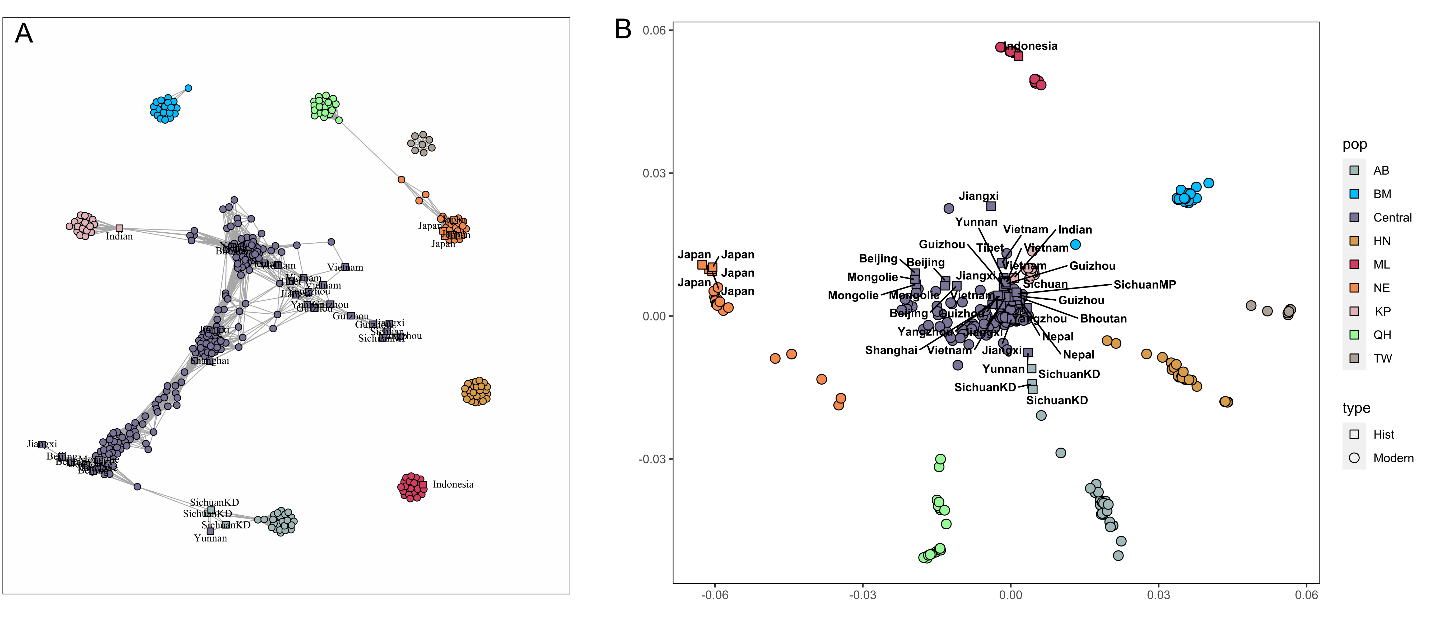


**Figure S3. Population structure inferred using NetView (A) and the non-metric multidimensional scaling (NMDS) clustering method (B)**. Historical samples are represented by square dots and labelled with corresponding location information. Different colors distinguish individuals sampled from the nine corresponding populations: AB (Aba), BM (Bome), CT (Central), HN (Hainan), ML (Malaysia), NE (Northeast), KP (Kashmir & Pakistan), QH (Qinghai), and TW (Taiwan). The figure reveals that most historical samples, along with modern central individuals, form a complex cluster, while several peripheral subspecies remain isolated.


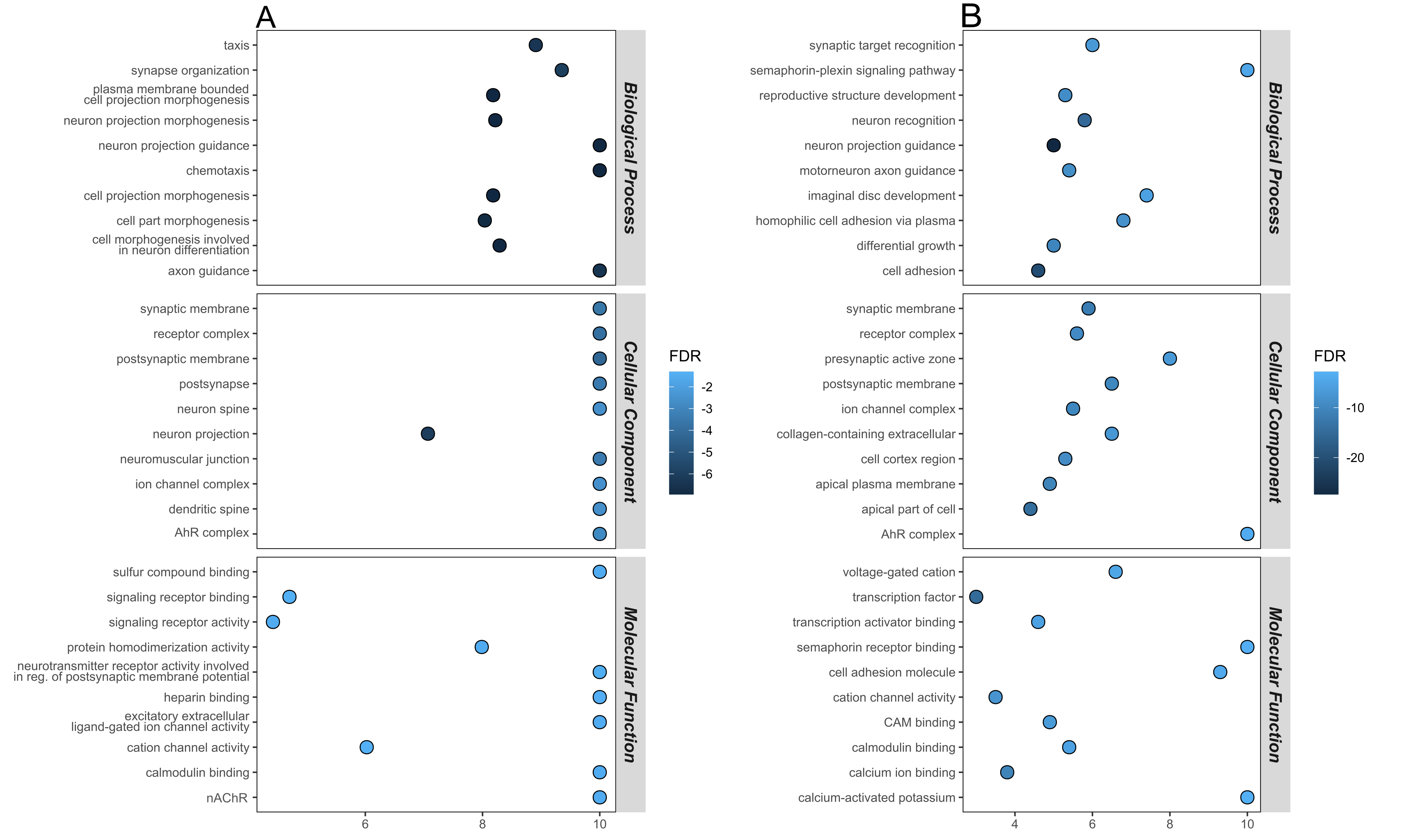


**Figure S4. Functional enrichment** **of genes targeted by selective sweep (A) and those spanning the outlier SNPs (B)**. Only the 10 most significant GO terms from each category are listed here, GO terms with fold enrichment (X-axis) > 10 were capped at 10 for clearer illustration. The complete list can be found in Table S5.

**
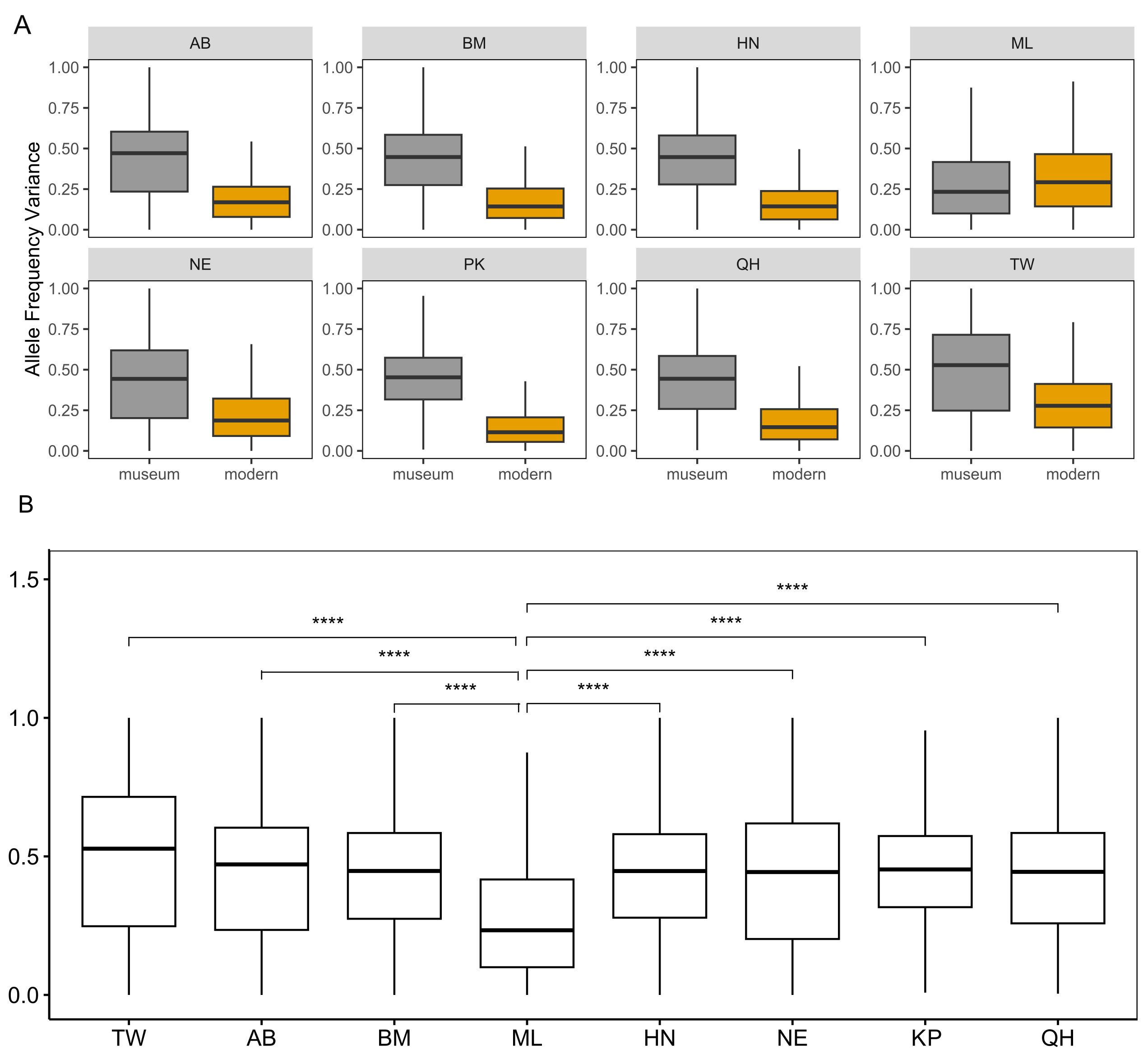
**

**Figure S5. Inter-population discrepancies** **for the outlier SNPs.** (A) the allele frequency variances between each contemporary peripheral population (subpanel) and the central population for the outlier SNPs, including both the historical central population (gray, left) and the modern central population (orange, right). The ML population is highlighted for its unique pattern in which the AFVs of the historical central population are significantly lower than other peripheral populations. (B) Allele frequency variance (Y-axis) between each peripheral population (X-axis) and the historical central population. *** denotes a statistically significant level with a p-value < 2.22e-16. Populations are labelled as: AB (Aba), BM (Bome), CT (Central), HN (Hainan), ML (Malaysia), NE (Northeast), KP (Kashmir & Pakistan), QH (Qinghai), and TW (Taiwan).


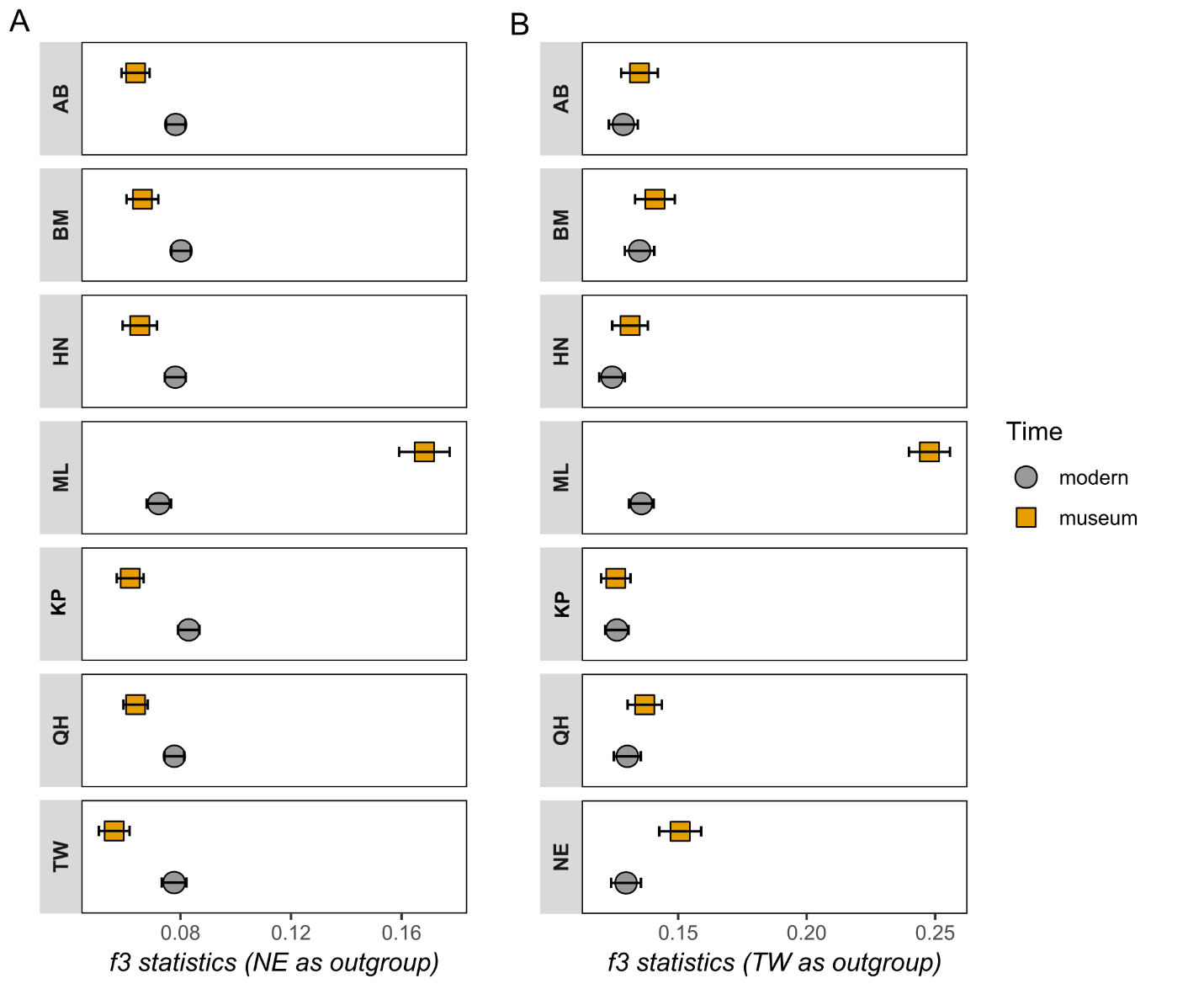


**Figure S6. The outgroup F3 statistics (X, Y; Z).** It was computed as follows: X denotes historical or modern central populations (color- and shape-coded as per the figure legend), Y represents peripheral populations (annotated in each strip), and Z corresponds to the outgroups (NE in Panel A and TW in Panel B). The higher the statistic, the more genetically similar X and Y are to one another. Populations are labelled as: AB (Aba), BM (Bome), HN (Hainan), ML (Malaysia), NE (Northeast), KP (Kashmir & Pakistan), QH (Qinghai), and TW (Taiwan).


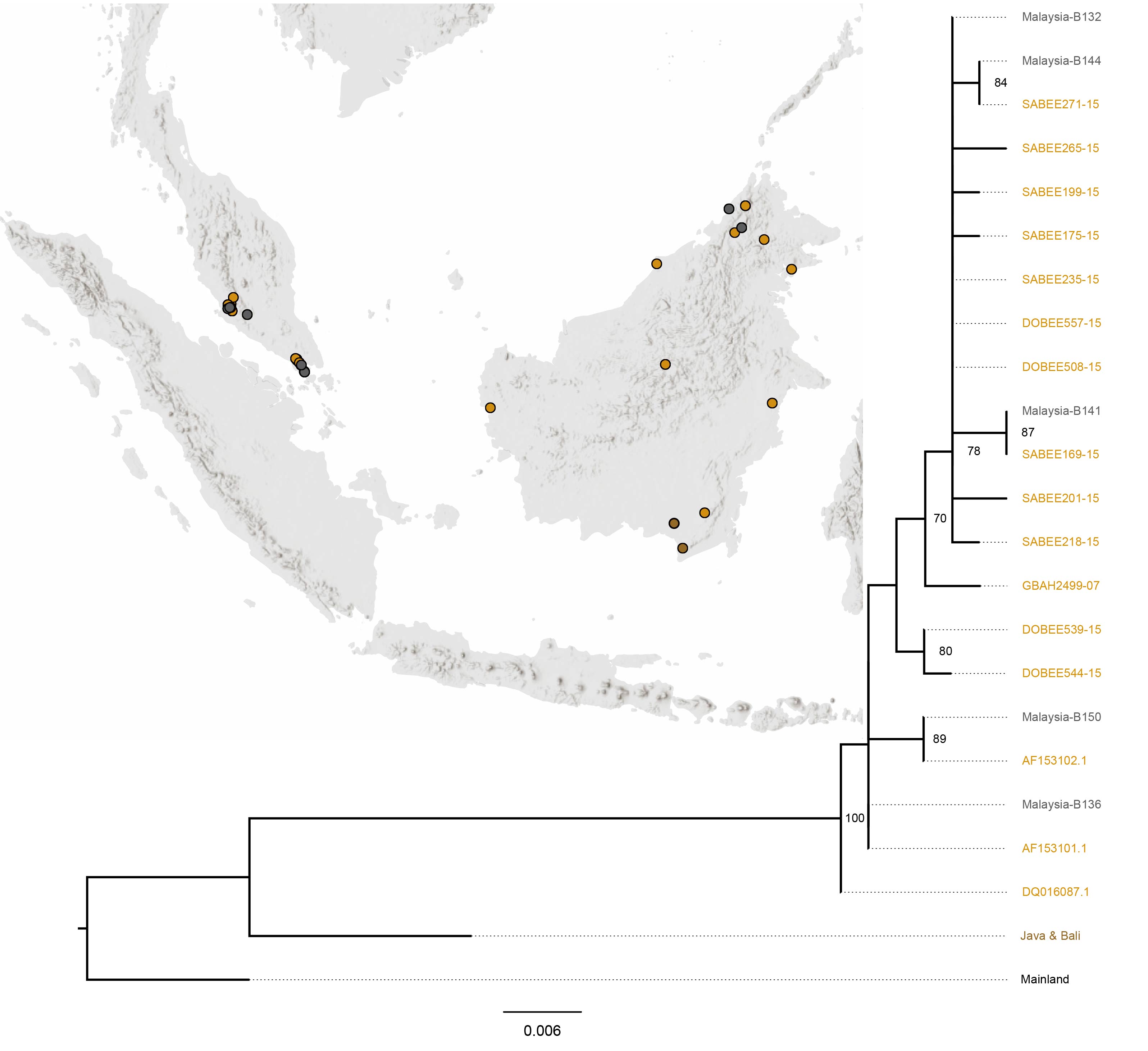


**Figure S7. Maximum-likelihood phylogeny of Malaysian *Apis cerana*.** Branch support values above 70 are shown at the corresponding internodes. The phylogeny is based on 22 COI haplotypes from 166 individuals. Colors on the map correspond to the geographic distributions of these haplotypes, with gray specifically representing the modern individuals used for whole-genome resequencing.

**
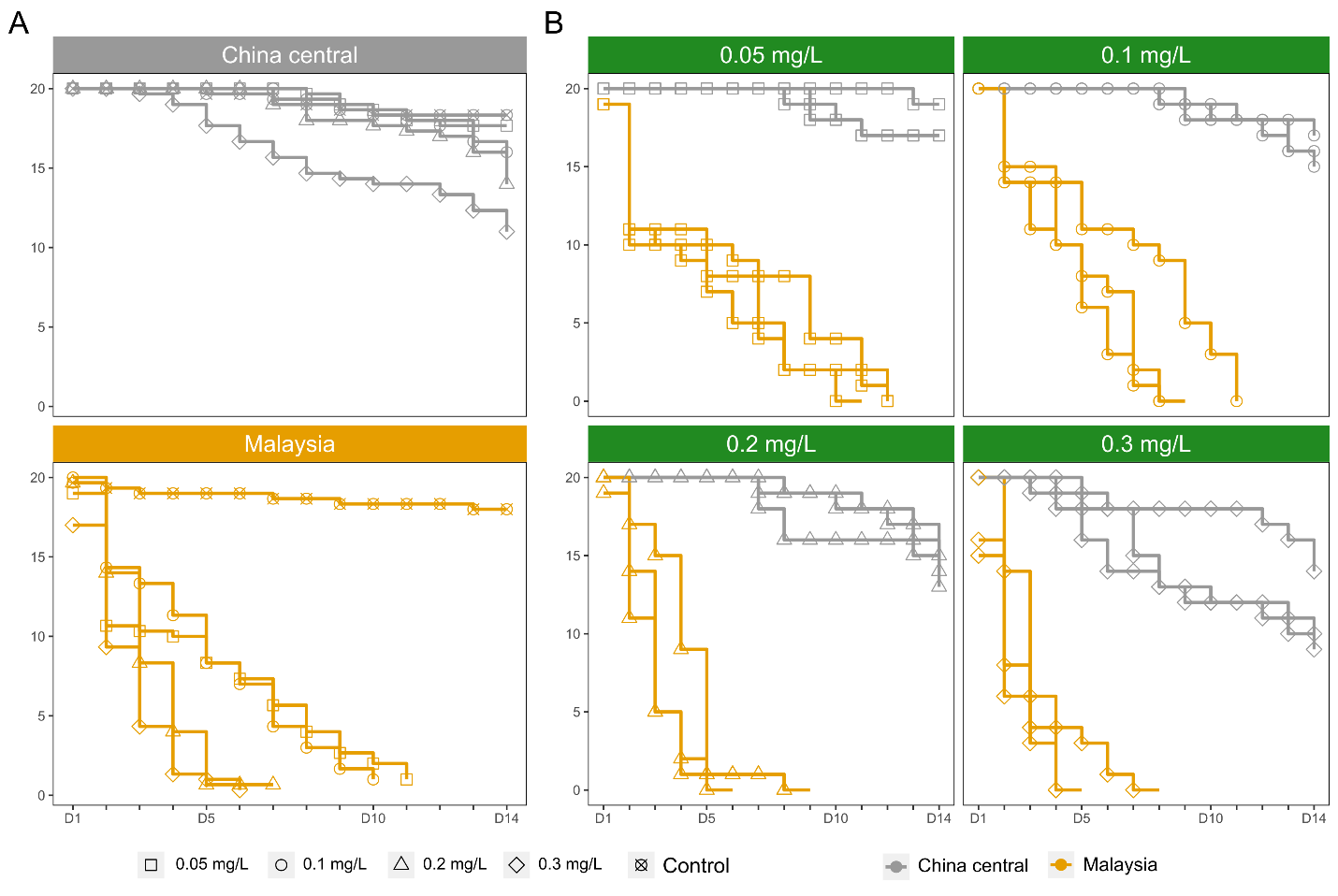
**

**Figure S8. The survival of honey bee workers after being exposed to Imidacloprid for 14 days.** The Y-axis of (A) represents the average number of surviving individuals across three biological replicates. The results of each biological replicate are shown separately in panel (B), for the four tests with different concentrations of Imidacloprid. Different dot shapes and line colors represent different concentrations and honey bee populations, respectively (detailed in the legend). The survival rate of Imidacloprid exposure test between the central China and Malaysia populations differs significantly (p value < 2e-16, Log Rank Test) in all but the control group (P value = 0.7).


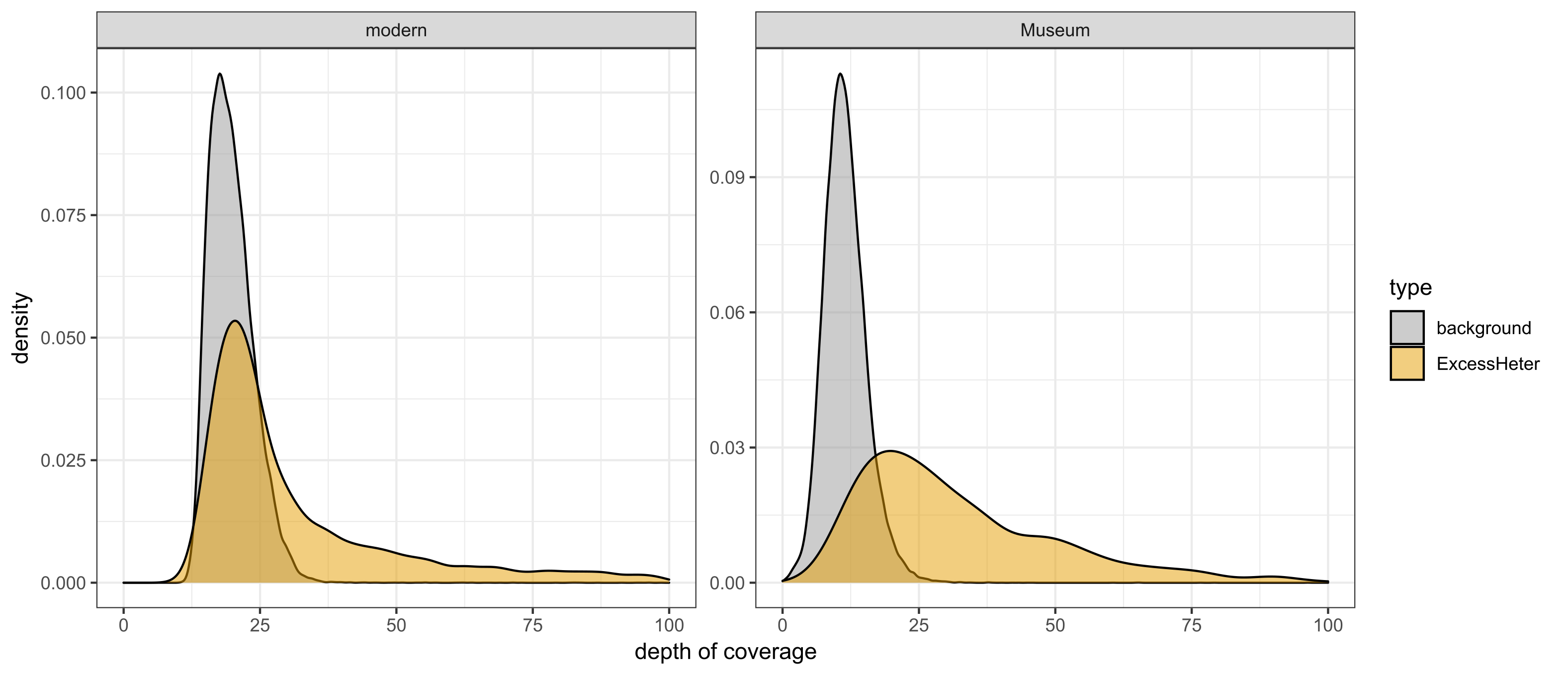


**Figure S9. Distribution of Sequencing Depth for Genomic SNPs.** The x-axis represents the depth of coverage, and the y-axis shows the distribution density. Each color represents a different SNP group with the “ExcessHeter” group specifically referring to SNP sites that exhibited a significant p-value in the exact test for excess heterozygosity, as determined during GATK annotation.


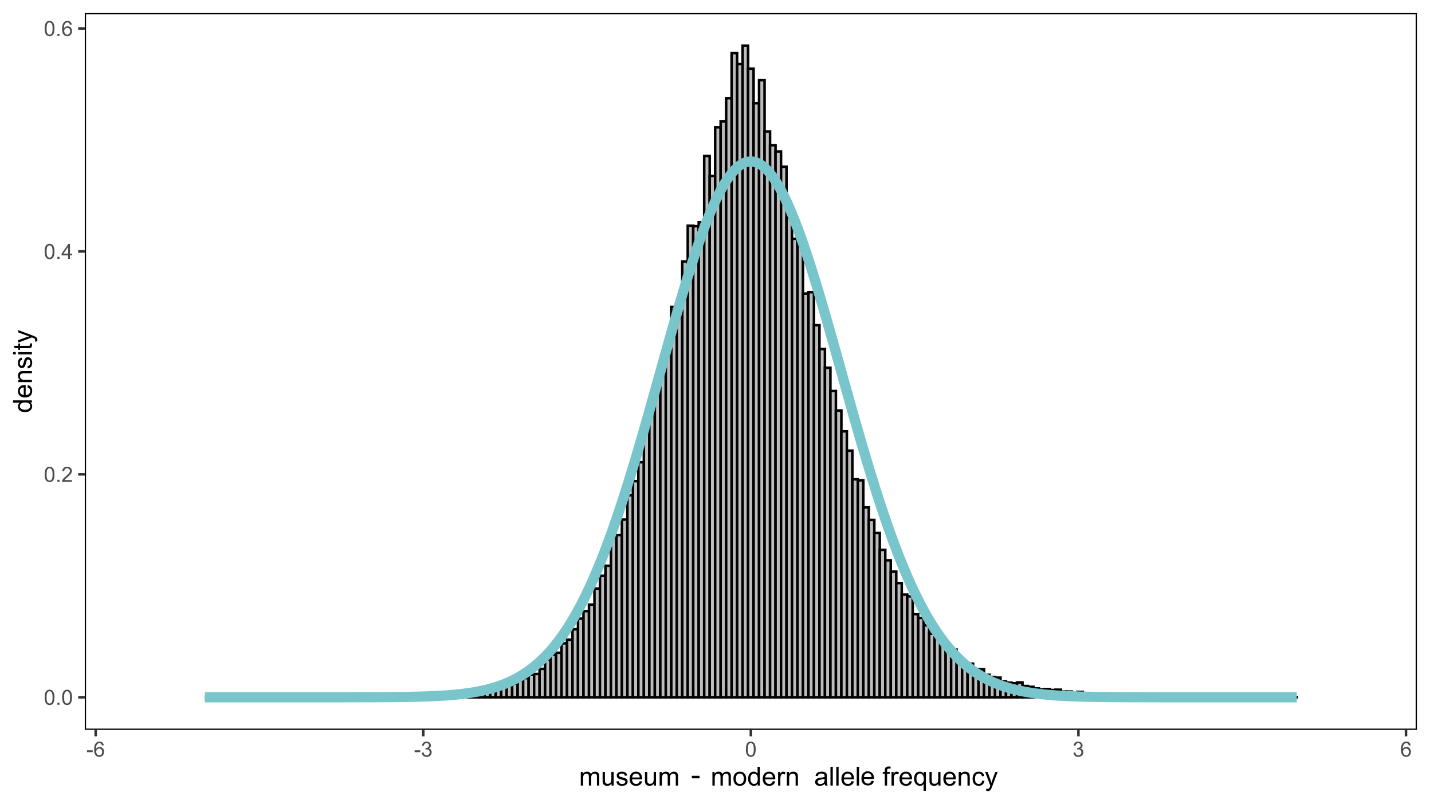


**Figure S10. Allelic frequency changes between the museum historical and the modern central populations for 278,075 SNPs.** Allele frequencies were subjected to angular transformation to stabilize variance, following Mikheyev et al. [30]. The blue line represents the standard normal distribution, with a mean of 0 and standard deviation (SD) of 0.83 that was calculated from the observed allele frequency differences between the historical and modern central population (𝑚𝑢𝑠𝑒𝑢𝑚 ― 𝑚𝑜𝑑𝑒𝑟𝑛).**REFERENCES**

1. Gilbert MTP, Moore W, Melchior L *et al.* DNA extraction from dry museum beetles without conferring external morphological damage. *PLoS ONE*. 2007; **2**: e272.

2. Cavill EL, Liu S, Zhou X *et al.* To bee, or not to bee? One leg is the question. *Mol Biol Evol*. 2022; **22**: 1868-1874.

3. Kapp JD, Green RE, Shapiro B. A fast and efficient single-stranded senomic library preparation method optimized for ancient DNA. *J Hered*. 2021; **112**: 241-249.

4. Ji Y, Li X, Ji T *et al.* Gene reuse facilitates rapid radiation and independent adaptation to diverse habitats in the Asian honeybee. *Sci Adv*. 2020; **6**: eabd3590.

5. Qiu L, Dong J, Li X *et al.* Defining honeybee subspecies in an evolutionary context warrants strategized conservation. *Zool Res*. 2023; **44**: 483-493.

6. Lindgreen S. AdapterRemoval: easy cleaning of next-generation sequencing reads. *BMC Res Notes*. 2012; **5**: 337.

7. Li H, Durbin R. Fast and accurate short read alignment with Burrows–Wheeler transform. *Bioinformatics*. 2009; **25**: 1754-1760.

8. Chen S, Zhou Y, Chen Y *et al.* fastp: an ultra-fast all-in-one FASTQ preprocessor. *Bioinformatics*. 2018; **34**: i884-i890.

9. Tarasov A, Vilella AJ, Cuppen E *et al.* Sambamba: fast processing of NGS alignment formats. *Bioinformatics*. 2015; **31**: 2032-2034.

10. Van der Auwera GA, Carneiro MO, Hartl C *et al.* From fastQ data to high‐confidence variant calls: the genome analysis toolkit best practices pipeline. *Curr Protoc Bioinformatics*. 2013; **43**: 11.10.1-11.10.33.

11. Hu J, Wang Z, Sun Z *et al.* NextDenovo: an efficient error correction and accurate assembly tool for noisy long reads. *Genome Bio*. 2024; **25**: 107.

12. Hu J, Fan J, Sun Z *et al.* NextPolish: a fast and efficient genome polishing tool for long-read assembly. *Bioinformatics*. 2019; **36**: 2253-2255.

13. Simão FA, Waterhouse RM, Ioannidis P *et al.* BUSCO: assessing genome assembly and annotation completeness with single-copy orthologs. *Bioinformatics*. 2015; **31**: 3210-3212.

14. Purcell S, Neale B, Todd-Brown K *et al.* PLINK: A tool set for whole-genome association and population-based linkage analyses. *Am J Hum Genet*. 2007; **81**: 559-575.

15. Manichaikul A, Mychaleckyj JC, Rich SS *et al.* Robust relationship inference in genome-wide association studies. *Bioinformatics*. 2010; **26**: 2867-2873.

16. Neuditschko M, Khatkar MS, Raadsma HW. NetView: A high-definition network-visualization approach to detect fine-scale population structures from genome-wide patterns of variation. *PLoS ONE*. 2012; **7**: e48375.

17. Dixon P. VEGAN, a package of R functions for community ecology. *J Veg Sci*. 2003; **14**: 927.

18. Liu S, Westbury MV, Dussex N *et al.* Ancient and modern genomes unravel the evolutionary history of the rhinoceros family. *Cell*. 2021; **184**: 4874-4885.e4816.

19. Korneliussen TS, Albrechtsen A, Nielsen R. ANGSD: Analysis of next generation sequencing data. *BMC Bioinformatics*. 2014; **15**: 356.

20. Chen H, Patterson N, Reich D. Population differentiation as a test for selective sweeps. *Genome Res*. 2010; **20**: 393-402.

21. Szpiech ZA, Hernandez RD. selscan: an efficient multithreaded program to perform EHH-based scans for positive selection. *Mol Biol Evol*. 2014; **31**: 2824-2827.

22. Quinlan AR, Hall IM. BEDTools: a flexible suite of utilities for comparing genomic features. *Bioinformatics*. 2010; **26**: 841-842.

23. Ge SX, Jung D, Yao R. ShinyGO: a graphical gene-set enrichment tool for animals and plants. *Bioinformatics*. 2019; **36**: 2628-2629.

24. Skoglund P, Mallick S, Bortolini MC *et al.* Genetic evidence for two founding populations of the Americas. *Nature*. 2015; **525**: 104-108.

25. Therneau TM, Grambsch PM. Modeling survival data: extending the cox model. *Statistics for Biology and Health*: Springer New York; 2000. 39-77.

26. Dobin A, Davis CA, Schlesinger F *et al.* STAR: ultrafast universal RNA-seq aligner. *Bioinformatics*. 2012; **29**: 15-21.

27. Pertea M, Pertea GM, Antonescu CM *et al.* StringTie enables improved reconstruction of a transcriptome from RNA-seq reads. *Nat Biotechnol*. 2015; **33**: 290-295.

28. Love MI, Huber W, Anders S. Moderated estimation of fold change and dispersion for RNA-seq data with DESeq2. *Genome Bio*. 2014; **15**: 550.

29. Zhao H, Sun Z, Wang J *et al.* CrossMap: a versatile tool for coordinate conversion between genome assemblies. *Bioinformatics*. 2013; **30**: 1006-1007.

30. Mikheyev AS, Tin MMY, Arora J *et al.* Museum samples reveal rapid evolution by wild honey bees exposed to a novel parasite. *Nat Commun*. 2015; **6**: 7991.
